# Thinking outside the mouse: emerging vertebrate models in biomedicine

**DOI:** 10.1242/bio.062293

**Published:** 2026-01-22

**Authors:** Sofia Ferreira-Gonzalez, Çağrı Çevrim, Aida Rodrigo Albors

**Affiliations:** ^1^ Centre for Inflammation Research, Institute for Regeneration and Repair, The University of Edinburgh, Edinburgh EH16 4UU, UK; ^2^ Department of Stem Cell and Regenerative Biology, Harvard University, Cambridge, MA 02138, USA; ^3^ Centre for Regenerative Medicine, Institute for Regeneration and Repair, The University of Edinburgh, Edinburgh EH16 4UU, UK; ^4^ Institute for Stem Cell Research, School of Biological Sciences, The University of Edinburgh, Edinburgh EH16 4UU, UK

**Keywords:** Unconventional models, Regeneration, Stem cells, Reproductive biology, Immunology, Ageing

## Abstract

The 2025 EMBO Workshop ‘Beyond the standard: Unconventional vertebrate models in biomedicine’ took place in Edinburgh, UK, in June 2025, and gathered a diverse group of researchers, veterinarians and animal technicians to explore the biological insights that can be unlocked from studying diverse, non-traditional vertebrates. This second iteration of the workshop focused on stem cells and regeneration, reproductive biology, immunology, ageing, and the latest technological advances and ongoing challenges in bringing non-model vertebrates to the forefront of biomedical research. The workshop also housed the first meeting for the growing spiny mouse research community.

## Teaser

**Summary:** Non-conventional vertebrate models open new research avenues in biomedicine, revealing natural mechanisms of regeneration, longevity, and disease resistance with promise for biomedical innovation.

## Introduction

Nature has produced an astonishing diversity of life, with many species possessing biological traits that extend far beyond what we typically observe in conventional model organisms and humans. Across the animal kingdom, some species can regenerate entire body parts, resist cancer, survive in extreme environments, or live far longer than expected for their size. These natural adaptations are not just curiosities; they provide natural solutions to ageing, disease resistance, tissue repair, and immune function. The axolotl redefines the limits of regenerative medicine, the spiny mouse heals skin wounds without scarring, and the naked mole-rat challenges our understanding of cancer biology and ageing. Such examples make clear that nature has already ‘experimented’ with a vast range of biological innovations over millions of years of evolution. By broadening our research focus to include unconventional animal models, we can tap into this immense source of previously unknown biology.

While working with unconventional models offers the potential for transformative insights, it also presents significant challenges. Progress is often slowed by limitations such as a lack of genetic tools, specialised facilities and husbandry expertise, limited training opportunities, and a relatively small research community with sparse infrastructure and funding for networking and collaboration. Recognising these gaps, we organised the EMBO Workshop ‘Beyond the standard: Unconventional vertebrate models in biomedicine’, focused on the interlinked areas of stem cells and regeneration, immunology, reproduction, and ageing, as well as tools and methods development. The workshop aimed not just to discuss the state of the field and emerging technologies, but also to foster new collaborations and build new bridges across research fields.

The workshop was organised by an international team of researchers including Aida Rodrigo Albors (Edinburgh University, UK), Sofia Ferreira-Gonzalez (Edinburgh University, UK), Çağrı Çevrim (Harvard University, USA), Ashley Seifert (University of Kentucky, USA), Mónica Sousa (iS3, Portugal), Peter Temple-Smith (Monash University, Australia), and Stuart Forbes (Edinburgh University, UK). It was held at the National Museum of Scotland, home to Dolly the sheep, the first mammal cloned from an adult cell. Dolly remains a powerful example of how an unconventional vertebrate can reshape scientific thinking and drive progress in biology and medicine.

**Fig. 1.**
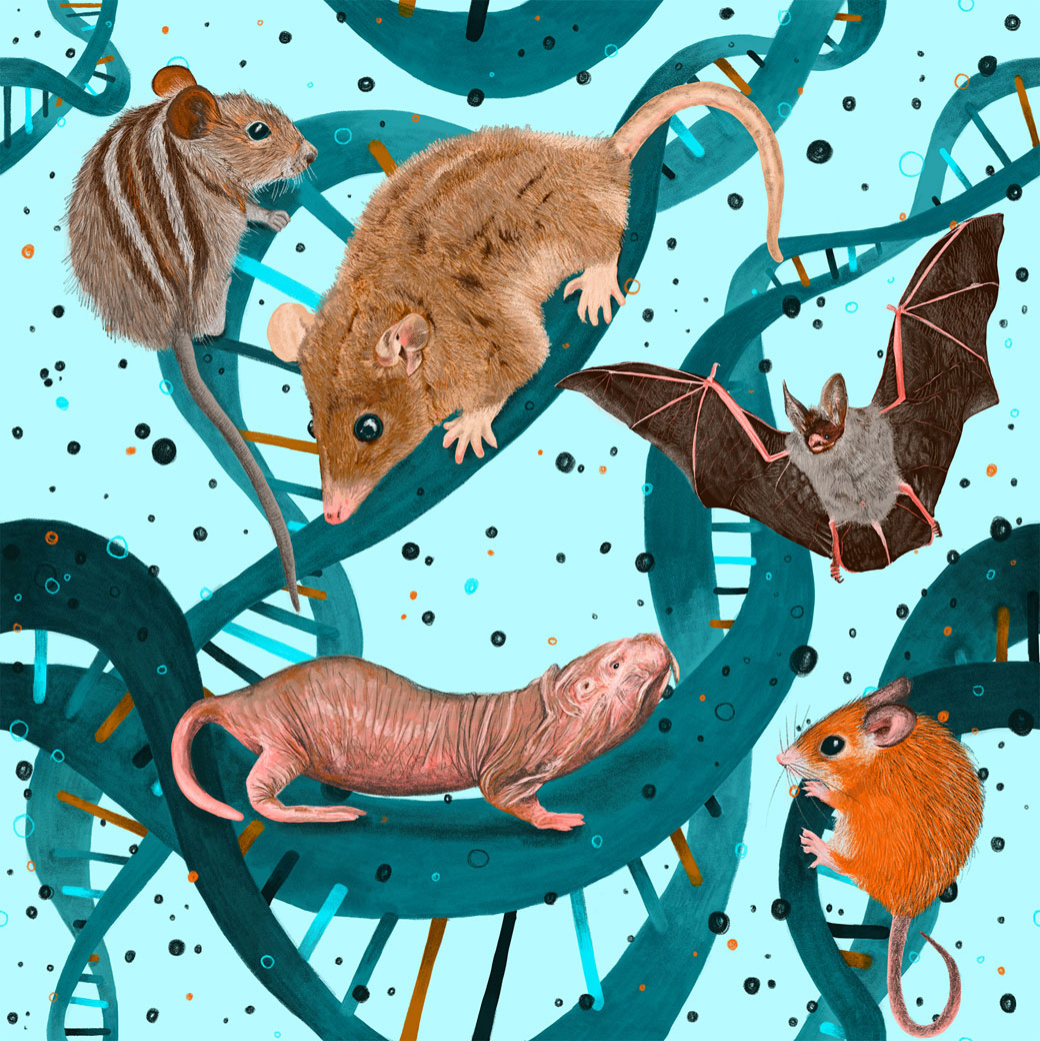
**Banner of the EMBO Workshop depicting only a few of the dozens of species represented at the meeting.** Drawing by Edinburgh-based illustrator Jennifer Colquhoun (The Illustrated Lab).

Throughout the workshop, presentations highlighted a fascinating diversity of species, from spiny mice and naked mole-rats to opossum, monito del monte, bats, Greenland sharks, and many more (Fig. 1). The vibrant mix of research fuelled lively and thought-provoking discussions throughout the event and well into the evenings.

## Spiny mouse satellite meeting

The spiny mouse (*Acomys spp*.) stands out as one of the few mammals capable of regenerating complex tissues, including skin and musculoskeletal tissue (Seifert et al., 2012), heart (Peng et al., 2021), kidney (Okamura et al., 2021), and even the central nervous system (Nogueira-Rodrigues et al., 2022). This remarkable prowess positions *Acomys* as an exceptional system for uncovering the mechanisms behind mammalian regeneration, with direct potential to improve human health. Beyond regeneration, *Acomys* is gaining traction in fields such as reproductive biology, being one of the only rodents known to menstruate (Bellofiore et al., 2018), and in behavioural neuroscience, due to its complex social behaviours (Fricker et al., 2022). Reflecting the rapidly expanding community of researchers, veterinarians, and research technical professionals interested in this unique mammal, we organised a spiny mouse satellite meeting preceding the workshop. Our goal was to unite the *Acomys* research community, facilitate collaboration, and coordinate efforts to establish *Acomys* as a widely recognised and valuable model organism.

Paving the way for that, David Thybert (University of East Anglia, Norwich, UK) and Ashley Seifert (University of Kentucky, Lexington, USA), described their collaborative work sequencing and annotating genomes across the *Acomys* clade, including *Acomys cahirinus*, *Acomys dimidiatus*, *Acomys percivali*, *Acomys kempi*, and *Acomys russatus*. Their comparative genomics data revealed a big surprise: most captive ‘*Acomys cahirinus*’ colonies are actually *A. dimidiatus* (Riddell et al., 2025). In response, the community agreed to adopt the name *A. dimidiatus* in all future publications. A forthcoming paper will introduce the *Acomys* clade-level genomics resource in detail.

In the spirit of open science, their chromosome-level assembly of the *A. dimidiatus* genome is already available through ENSEMBL–GCA_907164435.1 (mAcoDim1_REL_1905), providing a powerful resource for cross-species comparisons and already revealing fundamental principles of mammalian regeneration.

A major challenge of research on unconventional model organisms is the shortage of species-specific tools. Alex Araki (Artemis, USA) introduced Artemis, a new ‘focused research organisation’ aiming to expand the toolkit for *Acomys*, naked mole-rat, and tree shrew research with antibodies, complete genome annotations, and transgenic technologies. Recognising the importance of communication and resource-sharing, Sofia Ferreira-Gonzalez (Institute for Regeneration and Repair, The University of Edinburgh, Edinburgh, UK) presented SpinyHub, an upcoming web platform designed to centralise resources on *Acomys* biology. Inspired by platforms such as OpenHydra and AxoBase, SpinyHub will provide information on validated antibodies, husbandry, genomics, and more.

## Pushing boundaries: keynote advances in emerging models

The keynote presentations were truly groundbreaking, demonstrating how innovative research in non-traditional models is unlocking new directions in biology and medicine. Miki Ebisuya (Physics of Life TU Dresden, Dresden, Germany) introduced the ‘stem cell zoo’ (Lázaro et al., 2023), where identical cell types are derived from pluripotent stem cells of multiple mammalian species to enable direct, quantitative comparisons of developmental timing. Her work revealed that differences in biochemical reaction kinetics, rather than body size, explain much of the variation in developmental speed between species like mice and humans (Matsuda et al., 2025). This pioneering approach highlights how comparative stem cell systems can uncover the mechanisms of evolutionary change.

James Turner (The Francis Crick Institute, London, UK) used opossum as a model to explore sex chromosome evolution and regulation. His research demonstrated that marsupials employ unique molecular strategies for X chromosome inactivation and sex determination, challenging longstanding assumptions derived from traditional mammalian models (Leeke et al., 2025). Elena Gracheva (Yale School of Medicine, New Haven, USA) examined how 13-lined ground squirrels survive months of cold and dehydration through neural and metabolic adaptations that suppress thirst and enable prolonged torpor. Her findings offer broader insights into mammalian resilience and homeostasis under extreme conditions. Roberto Nespolo (Universidad Austral de Chile, Valdivia, Chile) examined the evolutionary and energetic foundations of hibernation in the monito del monte, uncovering traits that are informing new methods for organ preservation (Sabat et al., 2025). Collectively, these presentations demonstrated that embracing unconventional models propels innovation across developmental biology, genetics, physiology, and translational medicine.

## Expanding the toolbox: methodological innovations in non-conventional model organism research

Rapid technological advances empower scientists to go beyond the standard to address biological questions that were once out of reach. A consistent theme throughout the tools and technology session was the adaptation of techniques such as single-cell genomics, high-throughput sequencing, gene editing, and advanced imaging for use in species with unique biological traits. These innovations are being applied to a wide range of systems, from mapping neural circuits and regeneration in the axolotl (Barbara Treutlein, ETH Zurich, Basel, Switzerland) to studying immune system adaptations in bats and rodents, enabling comparative and evolutionary analyses at unprecedented scale and resolution. This session emphasized the ongoing efforts to refine and develop tools tailored for challenging organisms. Michael Hiller (LOEWE Centre for Translational Biodiversity Genomics, Frankfurt, Germany) with Bat1K (Morales et al., 2025) and David Thybert (University of East Anglia, Norwich, UK) with Rodent2K consortia demonstrated the power of comparative genomics and large-scale sequencing consortia to expand genomic resources for bats and rodents, respectively. Runshi Zheng (Institute for Regeneration and Repair, The University of Edinburgh, UK) showed how multimodal platforms integrating spatial transcriptomics with single-cell genomics can provide novel insights into tissue organisation and regeneration by enabling detailed cross-species comparisons.

Collaborative consortia and resource-sharing initiatives emerged as vital drivers of progress, accelerating the production of reference genomes and standardising analytical pipelines for the research community. As these new tools and strategies become increasingly accessible and experimental approaches continue to diversify, research on non-model vertebrates is rapidly evolving from a technically demanding niche into a source of transformative biological discovery.

## Rethinking immune diversity: novel models and ecological perspectives

The fields of immunology and metabolism are being transformed by unconventional vertebrate models, which reveal immune adaptations and metabolic traits that are hidden or absent in traditional laboratory settings. Stephan Rosshart (Friedrich-Alexander-Universität Erlangen-Nürnberg, Germany) introduced ‘wildlings’: laboratory mice that more closely mirror the immune system complexity of their wild counterparts, providing a more realistic framework for modelling human diseases (Runge et al., 2025). Further highlighting limitations of conventional lab-based approaches, Iris Mair (School of Biological Sciences, The University of Edinburgh, Edinburgh, UK) emphasised the ecological context by demonstrating how environmental variation outside the lab profoundly shapes immune system development and responsiveness in wild mice (Mair et al., 2024).

A recurring theme was the unique immune adaptations of mammals living in extreme environments. Gustavo Tiscornia (University of Algarve, Algarve, Portugal) presented evidence that *Acomys* display natural resistance to carcinogenesis, an adaptation apparently linked to the upregulation of multiple tumour suppressor genes (Vitorino et al., 2024 preprint). Huayun Hou (SickKids, Toronto, Canada) highlighted genomic and functional mechanisms that support survival and immune balance in *Acomys* under chronic water scarcity. These models offer important opportunities to study not only immunological diversity, but also the interplay between immunity, metabolism, and environmental stressors.

Translational applications were also a key focus: Jane Reznick [Cologne Excellence Cluster for Cellular Stress Responses in Aging-Associated Diseases (CECAD), Cologne, Germany] shared insights into the neotenous, stress-resistant cardiac physiology of naked mole-rats (Bundgaard et al., 2024 preprint); while Olivia Fleming (School of Biological Sciences, The University of Edinburgh, Edinburgh, UK) demonstrated how wood mice serve as a platform for field-testing novel anthelmintic vaccines, bridging the gap between laboratory research and real-world challenges in infectious disease.

Collectively, the session emphasised that moving beyond traditional laboratory models paves the way for biomedical innovation and fosters a richer, context-aware understanding of immune function and metabolic health.

## Breaking boundaries in reproductive biology: new models and new insights

Reproductive biology is increasingly embracing non-traditional mammalian models to address research gaps that classic laboratory species cannot fill. Menstruation exemplifies a critical process absent in common rodent models (Bellofiore et al., 2018). Peter Temple-Smith (Monash University, Melbourne, Australia) highlighted the value of *Acomys*, which menstruates naturally (Bellofiore et al., 2017). Çağrı Çevrim (2025) (Harvard University, Cambridge, USA) presented the development of the first transgenic lab mouse (*Mus musculus*) lines exhibiting menstruation. Camille Berthelot (Institute Pasteur, Paris, France) leveraged state-of-the-art technologies, including comparative single-cell transcriptomics and endometrial organoids to investigate the evolution of menstruation in primates (Brulport et al., 2024 preprint). Together, these novel models and methodologies set the stage to investigate menstrual biology, tissue regeneration and related pathologies such as endometriosis, providing much-needed experimental platforms to advance fundamental and translational research in women's health.

Another emerging focus was reproductive longevity. As the average age of parenthood rises, addressing age-dependent fertility decline has become increasingly important (Winkler et al., 2024). Lenka Gahurová (University of South Bohemia, České Budějovice, Czech Republic) highlighted the naked mole-rat as a remarkable species that maintains female fertility well into advanced age. This model offers a unique opportunity to uncover the biology underlying reproductive longevity, with promising implications for extending reproductive health in humans.

Driven by cross-species comparison and innovative technologies, it became clear that reproductive biology is gaining a richer, more nuanced understanding of mammalian reproduction. This progress heralds advances in fertility, menstrual health, and placental medicine, marking a decisive shift toward novel animal models to address longstanding gaps in women's health research.

## From scarring to healing: lessons in mammalian regeneration

Mammals with enhanced regenerative capacity offer unique opportunities to uncover strategies for activating regenerative programmes in species that typically heal with scarring, including humans. Regeneration is orchestrated through complex interactions between multiple resident cells, immune cells and the extracellular matrix (ECM), collectively determining whether healing results in scarring or true tissue restoration. This principle is vividly captured in spinal cord injuries; while laboratory mice and humans most often experience fibrosis and permanent functional loss, *Acomys* can naturally recover spinal cord function following complete transection (Nogueira-Rodrigues et al., 2022).

Mónica Sousa (iS3, Porto, Portugal) presented new evidence that a key difference lies in how the collagen type I-rich extracellular matrix deposited at the injury site is resolved: whereas this matrix persists in mice, *Acomys* resolve it and restart a regenerative programme that restores homeostasis and function. Expanding on this, Margherita Zamboni (Karolinska Institutet, Stockholm, Sweden) showed through longitudinal single-cell profiling that cells in the injured *Acomys* spinal cord return to homeostatic-like states, while mouse cells remain locked in a reactive state.

Reindeer provide another unique opportunity to directly compare determinants of fibrosis and regeneration, as their back skin scars following injury, but the antler velvet regenerates efficiently. Jeff Biernaskie (University of Calgary, Canada) found that although both skin and velvet fibroblasts exhibit a convergent injury response, only velvet fibroblasts revert to a homeostatic state that modulates the immune response and permits regeneration (Sinha et al., 2022). Highlighting the role of immune cells, Ashley Seifert (University of Kentucky, USA) described a tissue-resident macrophage subtype in *Acomys* that dampens the inflammatory response during ear pinna regeneration (Simkin et al., 2024), and Kerstin Bartscherer (Universität Osnabrück, Germany) is developing *Acomys*-like human macrophages to enhance stroke recovery. On the evolutionary front, Hanseul Yang (KAIST, South Korea) discovered that *Acomys* dorsal skin contains specialised ‘fracture lattice’, reminiscent of autotomy fracture planes in lizards and some salamanders. This fracture lattice forms a honeycomb-like pattern that enables rapid skin breakage and healing when pulled.

Collectively, these talks underscored the critical crosstalk between different cell types in determining regenerative outcomes and identified promising targets to shift pro-fibrotic cell states toward regeneration.

## Decoding longevity: lessons from unconventional ageing models

The field of ageing biology is being revolutionised through research on unconventional vertebrates that offer fresh perspectives on lifespan, healthspan, and disease resistance. Species such as naked mole-rats, bats, and Greenland sharks offer unique insights into the genetic, physiological, and ecological factors that shape the ageing process.

Ewan St John Smith (University of Cambridge, Cambridge, UK) highlighted mechanisms of tissue resilience and resistance to age-related degeneration in naked mole-rat brains, emphasising the crucial role of immune cells in preserving tissue function, alongside highlighting how increased gastrointestinal tract barrier integrity correlates with colitis resistance (Aguilera-Lizarraga et al., 2024). Arne Sahm (2024) (Leibniz Institute on Aging, Jena, Germany) provided genomic insights into the Greenland shark, a species renowned for its exceptional longevity and metabolic adaptations to stress-related injury. Dan Nussey (School of Biological Sciences, The University of Edinburgh, Edinburgh, UK) introduced the St Kilda Soay sheep project and demonstrated how longitudinal studies in wild populations reveal diverse ageing trajectories shaped by environmental and social factors – patterns often invisible in controlled laboratory settings (Nussey, 2025).

Philip Dammann (Universität Duissburg, Essen, Germany) explored how sociality influences longevity and healthspan in African mole-rats, shedding light on the impact of behaviour and social hierarchy on the ageing process within and across species (Sahm et al., 2021). Disease resistance emerged as another key theme. Emma Teeling (University College Dublin, Dublin, Ireland) revealed links between unique immune system profiles, extended healthspan, and reduced incidence of age-related diseases in bats (Athar et al., 2024). Ângela Gonçalves (DKFZ, Heidelberg, Germany) discussed cancer resistance pathways in Ansell's mole-rats, while additional presentations discussed comparative approaches to examine the molecular and physiological foundations of long-lived vertebrates.

By investigating this natural diversity of ageing strategies, the field is uncovering new genetic, environmental, and social determinants of longevity, with significant implications for understanding human healthspan and resilience to age-related diseases.

## A diverse and thriving research community

**Fig. 2.**
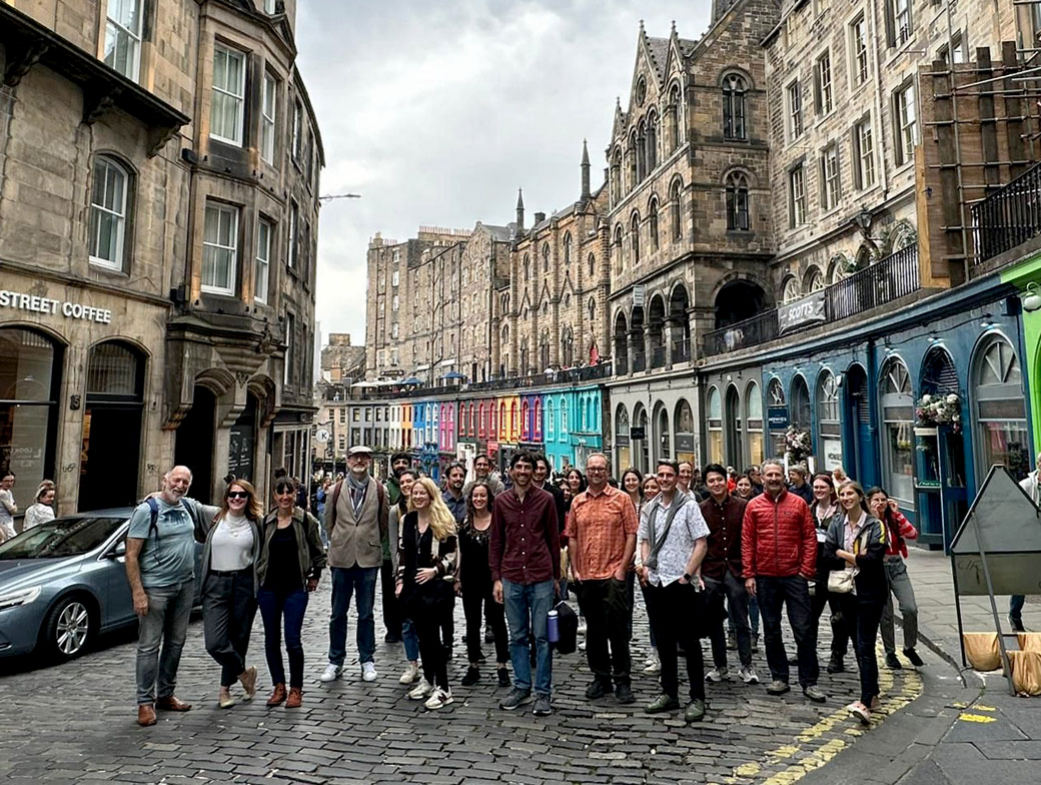
**Some participants of the EMBO Workshop out in the wild.** The streets of Edinburgh were a fitting background to this diverse and vibrant research community.

Since the inaugural EMBO Workshop on Unconventional Vertebrates in 2019, research communities working with captive and wild-caught mammals have grown significantly. This expansion was clearly reflected at the workshop, which welcomed participants from four continents. Early-stage principal investigators accounted for over 30% of the invited speakers, while PhD students and postdocs comprised 42% of the attendees. Notably, half of the workshop organisers were themselves early-career researchers, including two young PIs and a postdoc. These early-career researchers played a central role by sharing their work through short talks, flash presentations, and engaging poster sessions. They also actively participated in networking events such as the EMBO Women in Science discussion, city walk, and museum gala dinner (Fig. 2).

Outstanding contributions were recognised with a best talk prize awarded to Nikita Groot (University of St Andrews, St Andrews, UK) for their progress in establishing gene-editing techniques in bats to study language neurogenetics. The EMBO poster prize went to Leanne Donahue (Cornell University, Ithaca, USA), who showcased the link between cancer immunity and regenerative ability. The MacNeil poster prize (a special prize for undergraduate students sponsored by one of the organisers and named after his grandfather, who studied medicine in Edinburgh) was awarded to Isabel Ibeson (The University of Edinburgh, Edinburgh, UK) for her impressive research on breeding and parental behaviour in *Acomys*.

Together, these efforts reflect the dynamic, diverse, and collaborative community that continues to drive this field forward.

## Concluding remarks

There has never been a better time to venture beyond the standard. With high-quality genomes, stem cell-derived models, advanced single-cell genomics and transcriptomics we can now compare species comprehensively. New molecular tools will simplify the study of species that were once difficult to work with. We heard at the workshop how unconventional vertebrates are revealing new mechanisms for scarless regeneration, cancer resistance, and exceptional longevity, providing blueprints for tackling human challenges like tissue repair, ageing, and immune defence in ways that standard laboratory animals cannot offer.

To sustain this research momentum, strengthening collaborations and promoting open resource sharing is essential. Expanding centralised genomic and phenotypic databases and platforms like SpinyHub, as well as supporting consortia such as Bat1K and Rodent2K, can lower barriers for new researchers and support robust cross-species investigations. Investing in technical workshops, training and laboratory exchanges will provide early-career researchers with valuable experience in areas like specialised animal husbandry, gene editing, and advanced imaging. Extending these training initiatives to veterinarians and technical professionals is equally critical to ensure the effective management of research colonies and experiments – it truly takes a village. By investing in teamwork and training, this diverse and dynamic community is positioned to transform biological research, and drive innovation in biomedicine for years to come.
